# Gradual, but Not Sudden, Dose-Dependent Increase of ONJ Risk With Bisphosphonate Exposure: A Nationwide Cohort Study in Women With Osteoporosis

**DOI:** 10.3389/fendo.2021.774820

**Published:** 2021-12-09

**Authors:** Jung-Hyun Park, Min-Jeong Kwoen, Jae-Ryun Lee, Keun-Suh Kim, Hyo-Jung Lee, Jin-Woo Kim, Hyejin Lee

**Affiliations:** ^1^ Department of Oral and Maxillofacial Surgery, Research Institute for Intractable Osteonecrosis of the Jaw, College of Medicine, Ewha Womans University, Seoul, South Korea; ^2^ Department of Periodontology, Section of Dentistry, Seoul National University Bundang Hospital, Seongnam, South Korea; ^3^ Department of Family Medicine, Seoul National University Bundang Hospital, Seongnam, South Korea

**Keywords:** bisphosphonate, osteonecrosis of the jaw, osteoporosis, nationwide cohort, dose-dependent

## Abstract

**Background:**

A causal relationship of bisphosphonate (BP) exposure with osteonecrosis of the jaw (ONJ) has been reported; however, a definite dose-dependent risk remains to be elucidated beyond current vague recommendations of 4-year oral BP for ONJ risk increase.

**Objective:**

To identify the effect of bisphosphonate cumulative dose on ONJ development in women with osteoporosis.

**Methods:**

A retrospective cohort study was designed using the National Health Insurance Service—National Health Screening database of Korea. Females over the age of 50 were diagnosed with osteoporosis based on the International Classification of Diseases 10th revision (ICD-10) codes (M80, M81, and M82) with bisphosphonate prescriptions. The cumulative dose of bisphosphonate was calculated using defined daily doses (DDD) to provide an accurate BP cumulative effect on ONJ occurrence. Osteonecrosis of the jaw was identified using both ICD-10 codes and related procedure codes. The incidence rates of ONJ and hazard ratios were estimated according to the bisphosphonate cumulative dose.

**Results:**

Among 74,491 included subjects, 190 cases of ONJ were identified. The incidence rate substantially increased after BP cumulative dose over 1 year (25.75 for DDD < 365, which increased to 53.43 for 365 ≤ DDD < 730). Compared to subjects with a cumulative dose of DDD < 365, subjects with a cumulative dose of 365 ≤ DDD < 730 had 2.36-fold hazard for developing ONJ (p < 0.001).

**Conclusion:**

A bisphosphonate cumulative dose of more than 1 year had an increased risk of ONJ development. A gradual, but not sudden, dose-dependent increase in ONJ risk with BP exposure needs to be considered in providing the optimal BP treatment duration.

## Introduction

Osteoporosis, characterized by low bone mass and deterioration of the microenvironment of bone tissue leading to an increase in fracture risk, is a major public health problem, particularly in postmenopausal women (1). Bisphosphonates (BPs) are widely prescribed as a first-line treatment of osteoporosis based on robust evidence supporting the efficacy of BPs in decreasing the risk of vertebral, hip, and non-vertebral fractures (2).

Recently, there is increasing concern about prolonged BP therapy in patients with osteoporosis. In regard to efficacy, no additional benefit is found after a given period of time. However, the risk of complications such as medication-related osteonecrosis of the jaw (ONJ) and atypical femoral fracture increases due to the long skeletal retention time of BPs (3). The incidence of osteonecrosis of the jaw (ONJ) among patients with cancer and metastatic bone disease being treated with bisphosphonates is as high as 10%, which dictates that an understanding of the risk factors, preventative measures, means of early diagnosis, and treatment is critical, despite ONJ occurring in the clinical setting of intravenous bisphosphonates (4, 5). ONJ could occur in patients with underlying comorbidities and not receiving BP treatments. There are other causes associated with a higher risk of ONJ, such as multiple dental extractions. ONJ can be non-invasively studied, imaged utilizing bone scintigraphy (6). Organizations have tried to provide guidance on the duration of BP therapy (2, 7, 8). The Task Force of the American Society for Bone and Mineral Research suggested that after 5 years of oral BP or 3 years of intravenous BP, women with osteoporosis should be reassessed as whether to continue therapy (2). These suggestions were proposed mainly with respect to BP efficacy and reducing the risk of vertebral fracture.

Because ONJ is a potential harm of BP therapy, it is necessary to determine how ONJ development may affect the duration of the therapy to provide the optimal treatment duration with a risk/benefit perspective (2). Although the Task Force reviewed both efficacy and safety data for BP treatment of osteoporosis, evidence concerning the duration of BP therapy on ONJ development was limited. The variability and inconsistency in the study designs and diagnostic criteria and the rarity of the disease have made the cumulative effect of BPs unclear (9).

Guidelines from the American Association of Oral and Maxillofacial Surgeons described that BP exposure over 4 years had a higher risk for ONJ (10). They recommended an education of the potential risks of ONJ at the initiation of treatment in patients who are likely to exceed 4 years of treatment, and a drug holiday before any type of dental surgery in patients exposed to BP for more than 4 years. These recommendations were based on the study by Lo et al. (11) which evaluated the Kaiser Permanente database and found that the prevalence of ONJ in patients receiving oral BP therapy was 0.1%, which increased to 0.21% among patients with greater than 4 years of oral BP exposure. After the study publication, however, several population-based studies reported different results about BP exposure duration. In these studies, ONJ risk increased markedly before 4 years of BP treatment (12–15). Regardless, the guidelines of most organizations have stated that a BP exposure duration of more than 4 years has a higher risk of ONJ (10, 16–19).

Previous ONJ epidemiologic studies have attempted to uncover the causal relationship between BP duration and ONJ development but had limitations in their study designs. Different ONJ operational definitions account for significant errors of incidence estimations (20). In addition, in most of the studies assessing the risk of ONJ, the duration of BP exposure was used to determine the cumulative effect of BPs. However, duration of BP exposure, a period from BP start to the last BP intake reported by either patients or claim data, is an improper marker to describe skeletal BP accumulation, considering various drug types, formulations, dosages, and frequency. To provide an accurate BP cumulative effect on ONJ development, standardization of the cumulative dose is needed. Defined daily doses (DDDs), the usual dose that a subject would receive per day for osteoporosis, can be used to standardize different dosages for different drugs (21).

To provide the optimal treatment duration from a risk/benefit perspective, the BP exposure duration as an ONJ risk would need to be further assessed through a well-designed longitudinal cohort study. This nationwide cohort study identified the cumulative effect of BP on ONJ development in women with osteoporosis. The incidence rates of ONJ and hazard ratios were estimated according to the BP cumulative dose using DDDs to standardize across different dosages for the drugs.

## Methods

### Data Source

Our retrospective cohort study was designed using the National Health Insurance Service—National Health Screening (NHIS-HEALS) database of Korea. The NHIS provides a research database with the purpose of making useful data available to health researchers (22). The NHIS is the sole insurance provider in Korea, controlled and supported by the Korean government, and applies to almost 97% of Koreans. The Medical Aid program, which is administered by the NHIS, supports the remaining 3% of the population. Subscribers of the NHIS are encouraged to receive standardized health examinations once every 2 years. The NIHS-HEALS cohort consisted of random samples representing approximately 510,000 individuals aged 40–79 years, which is equal to 10% of the total population who attended the medical health examinations (22). The NIHS-HEALS database contains information about demographics and socioeconomic status, as well as medical database including diagnoses, prescriptions, and treatment modalities between January 2002 and December 2015. This study was approved by the Institutional Review Board (No. X-2004/606-905). Informed consent was waived because retrospective anonymized data were used.

### Study Population

**Figure 1:**
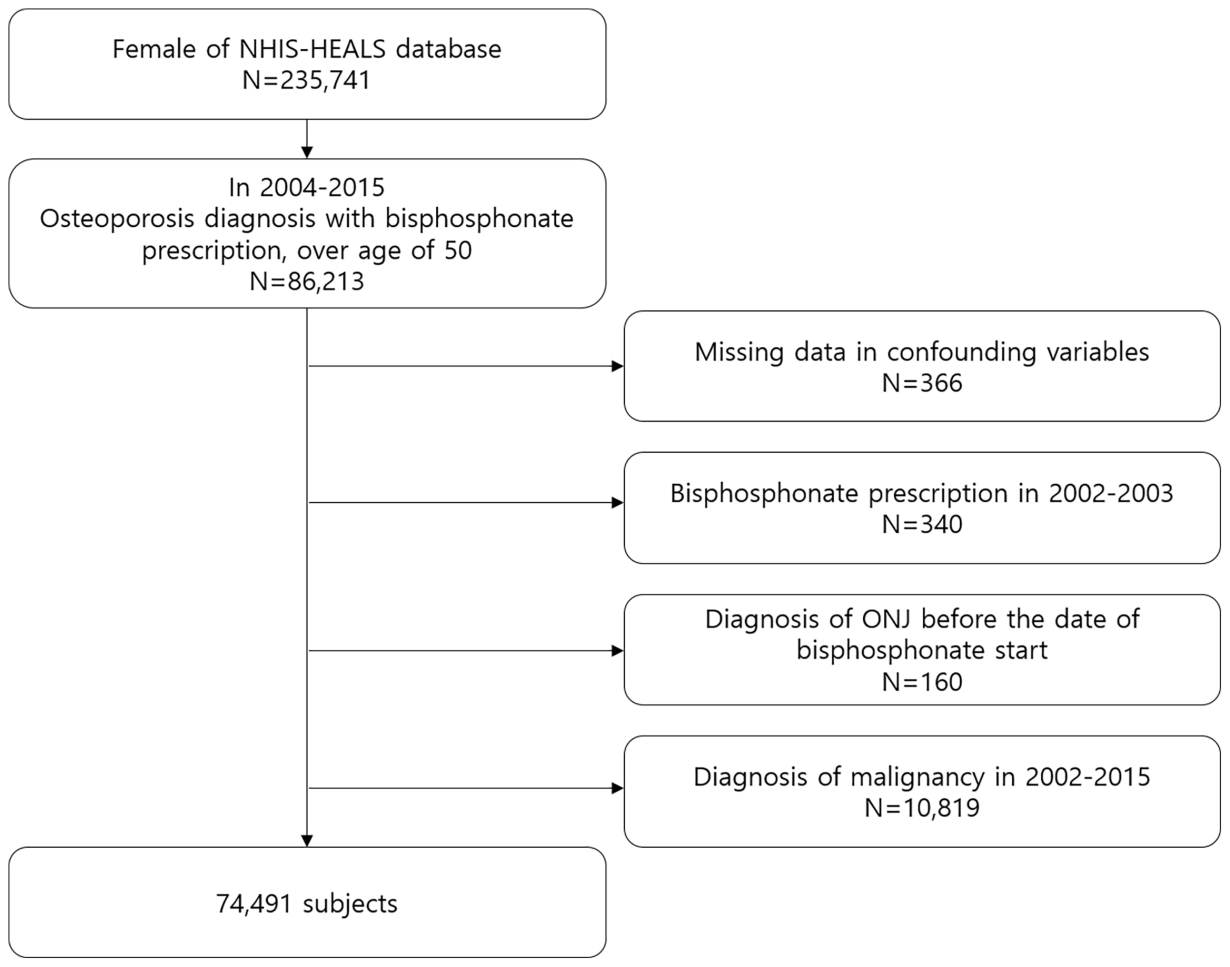
Flow chart of subject inclusion.

Based on the NHIS-HEALS database, we identified females over the age of 50 with BP prescriptions who were diagnosed with osteoporosis based on the International Classification of Diseases 10th revision (ICD-10) codes (M80: osteoporosis with pathological fracture; M81: osteoporosis without pathological fracture; M82: osteoporosis in disease classified elsewhere). Bisphosphonate exposure was identified *via* the prescription records in the database. Pamidronate, alendronate, risedronate, ibandronate, and zoledronate with all dosage forms and formulations were included in this study. Because this study aimed to identify the effect of cumulative dose on ONJ development, the first 2 years (from January 2002 to December 2003) were considered as a washout period to ensure new users of BP. The index date was the date of commencement of BP therapy. Subjects with a diagnosis of ONJ before the index date were excluded. We also excluded subjects who had malignant neoplasm using ICD-10 codes (C00–C97) between 2002 and 2015. Finally, 74,491 subjects for analysis were included. **Figure 1** shows the flowchart of subject inclusion. The follow-up period was done from the index date until ONJ occurred, the subject died, or December 2015, whichever occurred first.

### ONJ Definition

The primary outcome was the occurrence of ONJ. The definition of ONJ was one or more claims of ICD-10 codes (M87.1: osteonecrosis due to drugs; K10.2: inflammatory condition of jaws) with one of the related procedure codes. The date of ONJ diagnosis was defined as the first claim date of an ONJ diagnosis code.

### Cumulative Dose of Bisphosphonates

For each drug, the dose was calculated using defined daily doses (DDD). One DDD is the recommended dose of a drug (21). One DDD also equals the usual dose that a subject would receive per day for osteoporosis treatment. The cumulative dose of BP was presented by means of DDD. It was calculated by multiplying the number of prescribed drugs with the amount of drug divided by DDD. For example, the DDD for alendronate is 10 mg. For a subject who was prescribed 100 tablets of alendronate at 70 mg, the cumulative dose is 700 DDDs (100 × 70/10 = 700). This can be interpreted as the subject being exposed to alendronate for 700 days (13). For each subject, the cumulative dose was calculated by total prescribed DDD between the index date and the last follow-up date. The subjects were grouped by the calculated cumulative dose as DDD < 365, 365 ≤ DDD < 730, 730 ≤ DDD < 1,095, 1,095 ≤ DDD < 1,460, and DDD ≥ 1,460.

### Confounding Variables

The baseline date was defined as 1 year prior to the date of ONJ diagnosis in subjects with ONJ development or 1 year prior to the last follow-up date in subjects without ONJ development. These different definitions according to ONJ diagnosis are to ensure the baseline characteristics as factors that could affect ONJ development. Confounding variables were identified *via* following criteria for 1 year from the baseline date of each subject.

Economic status was classified into two groups (those covered by Medical Aid and others). Hypertension, diabetes, hyperlipidemia, myocardial infarction, stroke, and anemia were defined using ICD-10 codes for hypertension (I10, I15), diabetes mellitus (E10, E118, E119, E13, E149), hyperlipidemia (E78), myocardial infarction (I21, I22), stroke (I60–63), or anemia (D46, D50–53, D55–64, D74). The NHIS-HEALS dataset includes information on disability obtained from the Korean National Disability Registry, which includes physical impairment, brain impairment, visual impairment, hearing impairment, linguistic impairment, and mental impairment (23). Periodontal disease was defined as two or more of claims under the diagnosis of ICD-10 codes K052–056. Dentoalveolar surgery was defined as one or more claims of dental procedure codes, which involve surgical trauma to the jawbone and the need for bone remodeling (6). Steroid history was identified from prescription claims between the index date and the last follow-up date. Average steroid prescriptions per year were calculated by total prescription days divided by total follow-up years.

The bisphosphonate administration route was classified into two groups: the oral group, which refers to only oral BP intake, and the intravenous group, which refers to at least one intravenous BP injection. Because of the mixed use of BPs, the type of BP was classified into five groups considering drug potency (24): 1) zoledronate (at least one prescription of zoledronate), 2) risedronate (at least one prescription of risedronate without use of zoledronate), 3) ibandronate (at least one prescription of ibandronate without use of zoledronate, or risedronate), 4) alendronate (at least one prescription of alendronate without use of zoledronate, risedronate, or ibandronate), and 5) pamidronate (at least one prescription of pamidronate).

### Statistical Analysis

The baseline characteristics of the groups were compared using the chi-square test for categorical variables and the t-test or analysis of variance for continuous variables. To estimate ONJ incidence, the number of ONJ cases was divided by the sum of person-years in each dosage group. A 95% confidence interval (CI) for each incidence rate was computed using the Clopper–Pearson method. To determine hazard ratio, Cox proportional hazard regression was used with adjustment forage, economic status, disability, comorbidities, steroid prescription, BP type, BP administration route, and dental status. Sensitivity analysis was performed to investigate the robustness of the findings by regrouping of cumulative dosage as DDD < 365 and DDD ≥ 365, DDD < 730 and DDD ≥ 730, DDD < 1,095 and DDD ≥ 1,095, and DDD < 1,460 and DDD ≥ 1,460. All analyses were performed using R programming version 3.3.3 (The R Foundation for Statistical Computing, Vienna, Austria). All values were considered statistically significant if p-value < 0.05.

## Results

The mean age of included subjects was 70.2 years. Among 74,491 subjects, 57.1% was a cumulative dose of DDD < 365, 18.1% of 365 ≤ DDD < 730, 10.1% of 730 ≤ DDD < 1,095, 6.4% of 1,095 ≤ DDD < 1,460, and 8.3% of DDD ≥ 1,460. **Table 1** shows the baseline characteristics of the study population according to the cumulative dose of BP.

**Table 1:** Baseline characteristics of the study population and comparison of variables according to cumulative dose of bisphosphonate.

Characteristic	Cumulative dose					p-value
	DDD < 365	365 ≤ DDD < 730	730 ≤ DDD < 1,095	1,095 ≤ DDD < 1,460	DDD ≥ 1,460	
	(N = 42524)	(N = 13496)	(N = 7497)	(N = 4795)	(N = 6179)	
**Age**						
Mean (SD) - year	66.8 ± 9.0	69.9 ± 8.3	70.4 ± 7.8	70.9 ± 7.6	72.9 ± 7.2	<0.001
Median (IQR) - year	70 (14)	70 (12)	71 (11)	71 (10)	73 (10)	<0.001
Distribution - no. (%)						<0.001
50–59	6,789 (16.0%)	1,724 (12.8%)	756 (10.1%)	413 (8.6%)	280 (4.5%)	
60–69	13,699 (32.2%)	4,587 (34.0%)	2,557 (34.1%)	1,549 (32.3%)	1,539 (24.9%)
70–79	15,386 (36.2%)	5,466 (40.5%)	3,228 (43.1%)	2,193 (45.7%)	3,271 (52.9%)
≥80	6,650 (15.6%)	1,719 (12.7%)	956 (12.8%)	640 (13.3%)	1,089 (17.6%)
**Economic status - no. (%)**						<0.001
Medical aid	1,463 (3.4%)	557 (4.1%)	345 (4.6%)	230 (4.8%)	359 (5.8%)	
Others	41,061 (96.6%)	12,939 (95.9%)	7,152 (95.4%)	4,565 (95.2%)	5,820 (94.2%)
**Disability—no. (%)**						<0.001
No	37,533 (88.3%)	11,663 (86.4%)	6,474 (86.4%)	4,012 (83.7%)	5,030 (81.4%)
Yes	4,991 (11.7%)	1,833 (13.6%)	1,023 (13.6%)	783 (16.3%)	1,149 (18.6%)
**Comorbidities—no. (%)**						
Hypertension	21,684 (51.0%)	7,207 (53.4%)	3,940 (52.6%)	2,667 (55.6%)	3,606 (58.4%)	<0.001
Diabetes mellitus	11,325 (26.6%)	3,681 (27.3%)	2,024 (27.0%)	1,374 (28.7%)	1,730 (28.0%)	0.010
Dyslipidemia	20,913 (49.2%)	7,496 (55.5%)	4,282 (57.1%)	2,845 (59.3%)	3,741 (60.5%)	<0.001
Anemia	5,546 (13.0%)	1,983 (14.7%)	1,075 (14.3%)	713 (14.9%)	1,045 (16.9%)	<0.001
Myocardial infarction	351 (0.8%)	80 (0.6%)	38 (0.5%)	36 (0.8%)	42 (0.7%)	0.007
Stroke	3,008 (7.1%)	919 (6.8%)	516 (6.9%)	349 (7.3%)	467 (7.6%)	0.361
**Steroid use**						<0.001
No	13,407 (31.5%)	2,904 (21.5%)	1,313 (17.5%)	692 (14.4%)	714 (11.6%)
Yes	29,117 (68.5%)	10,592 (78.5%)	6,184 (82.5%)	4,103 (85.6%)	5,465 (88.4%)
**Average steroid prescription per year**						<0.001
0	13,407 (31.5%)	2,904 (21.5%)	1,313 (17.5%)	692 (14.4%)	714 (11.6%)
0 < days < 90	28,492 (67.0%)	10,344 (76.6%)	6,046 (80.6%)	3,971 (82.8%)	5,281 (85.5%)
90 ≤ days < 180	296 (0.7%)	116 (0.9%)	61 (0.8%)	55 (1.1%)	79 (1.3%)	
Days ≥ 180	329 (0.8%)	132 (1.0%)	77 (1.0%)	77 (1.6%)	105 (1.7%)	
**Type of bisphosphonate - no. (%)**						<0.001
Pamidronate	666 (1.6%)	82 (0.6%)	37 (0.5%)	13 (0.3%)	14 (0.2%)	
Alendronate	17,767 (41.8%)	2,906 (21.5%)	1,234 (16.5%)	627 (13.1%)	675 (10.9%)
Ibandronate	5,250 (12.3%)	1,898 (14.1%)	924 (12.3%)	552 (11.5%)	505 (8.2%)	
Risedronate	18,549 (43.6%)	8,431 (62.5%)	5,210 (69.5%)	3,526 (73.5%)	4,890 (79.1%)
Zolendronate	292 (0.7%)	179 (1.3%)	92 (1.2%)	77 (1.6%)	95 (1.5%)	
**Bisphosphonate administration route**						<0.001
Oral	37,862 (89.0%)	10,678 (79.1%)	5,769 (77.0%)	3,584 (74.7%)	4,662 (75.4%)
Intravenous	4,662 (11.0%)	2,818 (20.9%)	1,728 (23.0%)	1,211 (25.3%)	1,517 (24.6%)
**Dental status—no. (%)**						
Dentoalveolar surgery	5,227 (12.3%)	1,720 (12.7%)	937 (12.5%)	641 (13.4%)	776 (12.6%)	0.220
Periodontitis	6,786 (16.0%)	2,361 (17.5%)	1,370 (18.3%)	848 (17.7%)	1,074 (17.4%)	<0.001

DDD, defined daily dose.

**Figure 2:**
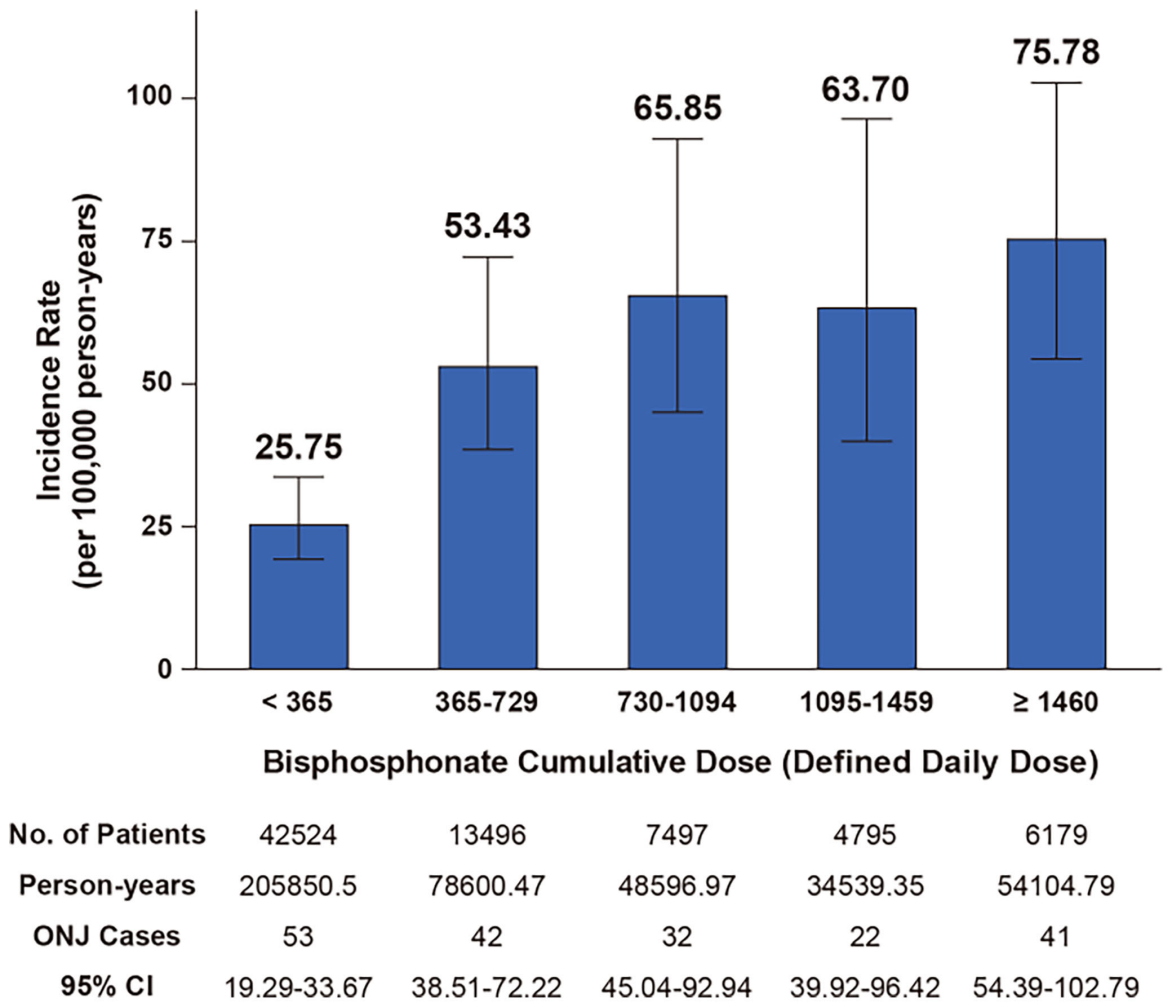
Incidence rate of ONJ according to cumulative dose of bisphosphonate.

In total, 190 cases of ONJ occurred and the incidence rate was estimated as 45.06 per 100,000 person-years (95% confidence interval, 38.88–51.94). **Figure 2** shows the incidence rate of ONJ according to the cumulative dose of BPs. The incidence rate per 100,000 person-years was 25.75 (95% confidence interval, 19.29–33.67) for DDD < 365, which increased to 53.43 (95% confidence interval, 38.51–72.22) for 365 ≤ DDD < 730. For DDD ≥ 730, the incidence rate was slightly increased to 63.70–75.78 as DDD increased, but it was not a dramatic increase.

**Figure 3:**
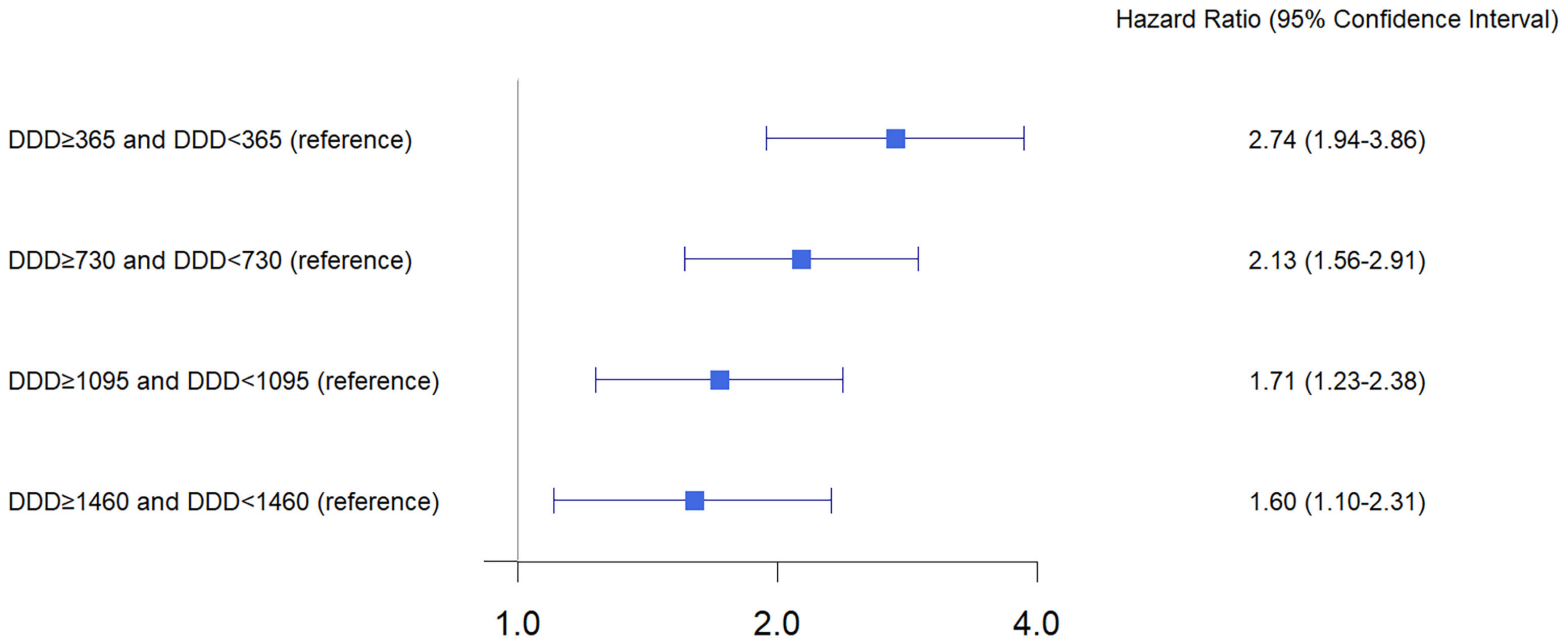
Sensitivity analysis for hazard ratio of ONJ according to cumulative dose.

**Table 2** shows hazard ratios for ONJ according to cumulative dose and other variables. Compared to subjects with a cumulative dose of DDD < 365, subjects with a cumulative dose of 365 ≤ DDD < 730 had a 2.36-fold hazard for developing ONJ (95% confidence interval, 1.53–3.58; p < 0.001). Subjects with a cumulative dose of 730 ≤ DDD < 1,095, 1,095 ≤ DDD < 1,460, and DDD ≥ 1,460 also had a significantly higher risk of ONJ development relative to subjects with a cumulative dose of DDD < 365, but the hazard ratios were slightly higher than the hazard ratio of subjects with a cumulative dose of 365 ≤ DDD < 730. The sensitivity analysis by regrouping of cumulative dosages (**Figure 3**) also showed that subjects with DDD ≥ 365 had a 2.74-fold increased risk of ONJ development as compared to DDD < 365 (95% confidence interval 1.94–3.86, p < 0.001). The hazard ratio of DDD ≥ 365 relative to DDD < 365 was higher than the hazard ratio of DDD ≥ 1460 relative to DDD < 1460 (hazard ratio, 1.06; 95% confidence interval 1.10–2.31, p < 0.001).

**Table 2:** Hazard ratios for ONJ development according to cumulative dose and other variables.

	Hazard ratio	95% CI	p-value
**Cumulative dose of bisphosphonate**			
DDD < 365	1.00 (reference)		
365 ≤ DDD < 730	2.36	1.56–3.58	<0.001
730 ≤ DDD < 1,095	3.12	1.97–4.93	<0.001
1,095 ≤ DDD < 1,460	2.85	1.70–4.80	<0.001
DDD ≥ 1,460	3.01	1.93–4.71	<0.001
**Age**			
50–59	1.00 (reference)	
60–69	1.40	0.65–2.98	0.390
70–79	1.92	0.92–4.02	0.083
≥80	2.15	0.97–4.76	0.058
**Economic status—no. (%)**			
Medical aid	1.00 (reference)	
Others	0.67	0.37–1.21	0.181
**Disability—no. (%)**			
No	1.00 (reference)	
Yes	0.32	0.18–0.57	<0.001
**Comorbidities—no. (%)**			
Hypertension	1.19	0.86–1.65	0.283
Diabetes mellitus	1.35	0.97–1.86	0.072
Dyslipidemia	0.74	0.54–1.02	0.067
Anemia	1.16	0.9–1.71	0.446
Myocardial infarction	1.51	0.37–6.13	0.565
Stroke	1.35	0.82–2.21	0.242
**Average steroid prescription per year**			
0	1.00 (reference)	
0 < days < 90	0.44	0.31–0.62	<0.001
90 ≤ days < 180	0.64	0.15–2.64	0.536
Days ≥ 180	4.07	2.14–7.73	<0.001
**Type of bisphosphonate—no. (%)**			
Pamidronate	1.00 (reference)	
Alendronate	0.80	0.19–3.27	0.752
Ibandronate	0.75	0.17–3.35	0.706
Risedronate	0.45	0.11–1.84	0.266
Zolendronate	0.45	0.04–5.38	0.530
**Bisphosphonate administration route**			
Oral	1.00 (reference)	
Intravenous	0.46	0.26–0.80	0.006
**Dental status—no. (%)**			
Dentoalveolar surgery	3.99	2.82–5.66	<0.001
Periodontitis	5.79	4.01–8.36	<0.001

CI, confidence interval; DDD, defined daily dose.

Adjusted for age, economic status, disability, comorbidities, steroid prescription, BP type, BP administration route, and dental status.

Subjects with disabilities had a lesser hazard ratio than those without disability (hazard ratio, 0.32; 95% confidence interval, 0.18–0.57; p < 0.001), indicating that subjects without disability had more ONJ development. Average steroid prescription days ≥1 80 had 4.07-fold hazard relative to no steroid prescriptions (95% confidence interval, 2.14–7.73; p < 0.001), but less risk was estimated in average steroid prescription days < 180. Intravenous BP administration had a decreased risk of ONJ development as compared to oral BP administration (hazard ratio, 0.46; 95% confidence interval 0.26–0.80, p = 0.006). Subjects with a history of dentoalveolar surgery (hazard ratio, 3.99; 95% confidence interval, 2.82–5.66; p < 0.001) or periodontitis (hazard ratio, 5.79; 95% confidence interval, 4.01–8.36; p < 0.001) had a significantly higher risk of ONJ development. There was no significant association between ONJ development and the following factors: age, economic status, comorbidities, and type of BP.

## Discussion

This study, using a nationwide database, estimated the incidence rate and hazard ratios of ONJ to identify the effect of BP cumulative dose on ONJ development in women with osteoporosis. The results of this study demonstrated that incidence of ONJ substantially increased after 1 year of BP cumulative dose. Subjects with more than 1 year of BP cumulative dose had a more than doubled risk of ONJ as compared to the subjects with less than 1 year BP of cumulative dose. In addition, the risk of ONJ in BP cumulative dose of 1–4 years was not distinctly less than the risk in BP cumulative dose of more than 4 years. Higher incidence was estimated in subjects with a BP cumulative dose of more than 4 years than in subjects with less than 4 years, but it was not a substantial increase. This is notable because previous studies reported a marked increase beyond 2–4 years of treatment duration (11–15).

To our knowledge, the effect of BP cumulative dose on ONJ development in osteoporosis women has not been evaluated in more than 70,000 subjects over a 10-year observation period. Several population-based studies have reported an association between ONJ development and BP exposure duration for osteoporosis treatment. Lapi et al. evaluated users of oral BPs prescribed for the secondary prevention of osteoporotic fractures using the Italian claims database (25). The authors reported a longer BP duration showing a trend toward an increased risk of ONJ, although they failed to show statistical significance due to the small sample size. Two studies by Lin et al. and Chiu et al. examined ONJ risk only among alendronate users based on the Taiwan claims database (15, 26). Lin et al. reported that patients who had received higher cumulative doses did not have an increased ONJ risk and concluded that there was no dose–response relationship between alendronate and ONJ (26). On the contrary, Chiu et al. demonstrated that the risk of ONJ began after 1 year of alendronate initiation and cumulative incidence of ONJ progressively increased with longer durations of treatment (15). Using the Korean claims database, Kwon et al. demonstrated that increased risk of ONJ as cumulative duration of BP exposure increased (12). Another study using the Korean claims database by Jung et al. reported that over 2 years of cumulative dose was associated with an increased risk of ONJ development (13). Aforementioned previous studies, however, included smaller numbers of individuals or only alendronate users and used the duration of BP exposure, a period from BP start to the last BP intake, rather than cumulative dose to determine the cumulative effect of BPs. In addition, the studies did not evaluate the risk beyond 2 years of BP cumulative dose. In the present study, the effect of BP cumulative dose on ONJ development was evaluated in more than 70,000 subjects and the risk of BP cumulative dose over 4 years was examined. In addition, to increase reliability of the determination of ONJ, a stricter operational definition than previous studies was used. A positive predictive value of ICD-10 codes in the ONJ identification has been reported low (20, 27), so ONJ was identified using both diagnosis and related procedure codes in this study.

Guidelines of organizations described patients with an oral BP treatment period of more than 4 years that had markedly increased the risk of ONJ (10, 16). They recommended a drug holiday before dental surgery in patients who had been exposed with BP for more than 4 years, but not in patients who had been exposed with BP for less than 4 years. However, according to our results, the BP cumulative dose of 1–4 years had a similar risk of ONJ as a BP cumulative dose of more than 4 years, so the guidelines may need to be reconsidered. Over-suppression of bone remodeling by BP is thought to be directly associated with ONJ development (10, 28). When BP administration is prolonged, the drug efficacy decreases and the increase in bone mineral density is limited. Likewise, the risk of ONJ substantially increases as BP is administered, but an additional risk increase may be limited after a given period of time.

Our result of the increased risk of ONJ during the average steroid prescription days was over 180 days per year, whereas short-term use of steroids (less than 180 days per year) was not a significant risk factor. Previous studies described that steroids are associated with an increased risk for ONJ (10, 16, 28). Long-term steroid use combined with BP was reported to increase ONJ risk because of destruction of the microenvironment of hard tissue (29). In the study by Tennis et al., steroid use over 90 days was associated with a doubling in the incidence of ONJ compared with use of less than 90 days or no steroid use in the cancer cohort (30). However, they could only find the association in the cancer cohort because the number of ONJ cases was limited in the osteoporosis cohort. Our results support that long-term steroid use may be associated with an increased risk of ONJ in osteoporosis patients as well.

Recent studies reported that the route of BP administration was a significant risk factor and that intravenous BP usually showed a higher incidence than oral BP (10). On the contrary, our results demonstrated that intravenous BP administration had a decreased risk of ONJ occurrence as compared to oral BP administration. This may be attributed to the calculation of the BP cumulative dose by means of DDD. Because DDD is the recommended dose of a drug that a subject would receive per day for osteoporosis (21), a similar pharmacologic effect can be anticipated in both intravenous and oral administration when BP is used at the recommended dosage. If the BP dose is standardized as DDD, there may be no risk differences in the administration route of BP in ONJ occurrence. The prescription bias of clinicians also might contribute to the higher ONJ risk of oral BP. There may be a tendency to prescribe oral BP in patients having dental risks because previous consensus-stated intravenous BP had an increased risk of ONJ (10, 16, 28).

This study has several limitations. First, due to the nature of the database, only claim data were used to define ONJ cases. Osteonecrosis of the jaw cannot be confirmed by medical records or direct oral examination, and severity of ONJ cannot be assessed. Second, there may be confounders not included in this study which can affect ONJ development. No adjustment was made for behavioral factors such as smoking and alcohol consumption because of the limited accessibility of the data used. Third, the study subjects only included the Korean population, and results may be different in other races/ethnicities as aforementioned. However, this study has the following strengths. First, we used large-scale nationally representative data that have been tracked for quite a long time to uncover the effect of BP cumulative dose on ONJ occurrence. Second, reliability of the data has increased by applying strict criteria to the determination of ONJ, which was identified using both diagnosis and related procedure codes. Third, to provide an accurate BP cumulative effect on ONJ occurrence, cumulative dose was calculated using DDD instead of using duration of BP therapy (a period from BP start to the last BP intake).

In conclusion, the overall incidence of ONJ of BP therapy in women with osteoporosis is low, but the BP cumulative dose of more than 1 year had an increased risk of ONJ development. The risk of ONJ in the BP cumulative dose of 1–4 years was not distinctly less than the risk in the BP cumulative dose of more than 4 years. A gradual, but not sudden, dose-dependent increase in ONJ risk with BP exposure needs to be contemplated to provide the optimal BP treatment duration.

## Data Availability Statement

Publicly available datasets were analyzed in this study. These data can be found here: National Health Insurance Data Sharing Service, https://nhiss.nhis.or.kr/bd/ab/bdaba000eng.do.

## Ethics Statement

The studies involving human participants were reviewed and approved by the Institutional Review Board of Seoul National University Bundang Hospital (No. X-2004/606-905). Written informed consent for participation was not required for this study in accordance with the national legislation and the institutional requirements.

## Author Contributions

J-HP contributed to the conception, design, and interpretation and drafted the manuscript. J-RL contributed to the data acquisition and analysis and critically revised the manuscript. M-JK, K-SK, and H-JL contributed to the conception and design and critically revised the manuscript. HL and J-WK contributed to the conception, design, data acquisition, and interpretation and critically revised the manuscript. All authors gave final approval and agree to be accountable for all aspects of the work.

## Funding

This work was supported by the National Research Foundation of Korea (NRF) grant funded by the Korean government (MSIT) (No. 2020R1A2C4001842).

## Conflict of Interest

The authors declare that the research was conducted in the absence of any commercial or financial relationships that could be construed as a potential conflict of interest.

## Publisher’s Note

All claims expressed in this article are solely those of the authors and do not necessarily represent those of their affiliated organizations, or those of the publisher, the editors and the reviewers. Any product that may be evaluated in this article, or claim that may be made by its manufacturer, is not guaranteed or endorsed by the publisher.
